# Serum ferritin levels in inflammation: a retrospective comparative analysis between COVID-19 and emergency surgical non-COVID-19 patients

**DOI:** 10.1186/s13017-021-00354-3

**Published:** 2021-03-08

**Authors:** Filippo Banchini, Gaetano Maria Cattaneo, Patrizio Capelli

**Affiliations:** Department of General Surgery, Guglielmo da Saliceto Hospital, Piacenza, Italy

**Keywords:** COVID, SARS-CoV-2, Ferritin, Sepsi, Surgery, Hepcidin, Transferrin, Iron

## Abstract

**Background:**

SARS-CoV-2 infection has spread worldwide, and the pathogenic mechanism is still under investigation. The presence of a huge inflammatory response, defined as “cytokine storm,” is being studied in order to understand what might be the prognostic factors implicated in the progression of the infection, with ferritin being one of such markers. The role of ferritin as a marker of inflammation is already known, and whether it changes differently between COVID and non-COVID patients still remains unclear. The aim of this retrospective analysis is to understand whether the inflammatory process in these two types is different.

**Methods:**

In this retrospective analysis, we compared 17 patients affected by SARS-CoV-2, who had been admitted between February and April 2020 (group A) along with 30 patients admitted for acute surgical disease with SARS-CoV-2 negative swab (group B). A further subgroup of Covid negative patients with leukocytosis was compared to group A.

**Results:**

In group A, the median (interquartile range) serum ferritin was 674 (1284) ng/mL, and it was double the cutoff (300 ng/mL) in 9 out of 17 (52%). The median (IQR) value of ferritin level in the total blood samples of group B was 231, and in the subgroup with leucocytosis, 149 (145). Group A showed a significantly higher ferritin median level compared to the entire group B (two-tailed Mann-Whitney test, *p* < 0.0001) as well as to the subgroup with leucocytosis (*p* < 0.0014).

**Conclusions:**

The role of iron metabolism appears to be directly involved in COVID infection. On the other hand, in the acute inflammation of patients admitted for surgery, and probably in other common phlogistic processes, iron modifications appear to be self-limited. However, our finding suggests the use of ferritin as a marker for COVID infection.

## Background

Coronavirus disease 19 (COVID-19) is a complex multisystemic disease whose pathogenesis is still being studied. Going by the clinical evidence, and the data reported in current literature, most COVID-19 patients develop an abnormal inflammatory response to the viral infection, leading to multiorgan failure and death (https://www.who.int/emergencies/diseases/novel-coronavirus-2019/question-and-answers-hub/q-a-detail/q-a-coronaviruses#:~:text=symptoms). The inflammatory process due to SARS-CoV2 infection may play a main role in the pathogenesis of multiple organ damage and be responsible for the dramatic outcome of COVID-19 patients. The early identification of COVID-19 patients with negative prognostic factors might be quite useful in the management strategy, in order to limit severe complications and eventual death. Inflammatory markers include white blood cell count, lactate dehydrogenase, C-reactive protein, fibrinogen, and D-dimer, which are commonly used in clinical practice to monitor the process of sepsis [1, 2]. In literature, it established that iron metabolism is involved in several pathogenetic disease mechanisms, including infections and various hematological and immunological disorders [3].

Recent data as reported in the current literature show that iron metabolism can undergo significant modifications which can be employed in predicting mortality even in patients admitted in intensive care units. Furthermore, serum ferritin has been recently cited as one of the indicators of mortality in COVID-19 patients [4]. Therefore, we decided to investigate serum ferritin as a marker of inflammation, comparing COVID-19 patients admitted into our Emergency Department for acute respiratory syndrome to non-COVID-19 patients which were hospitalized for acute surgical diseases in our Emergency Surgery Department. Our study aims to understand if serum ferritin could be used as an early laboratory marker to identify and classify COVID-19 patients.

## Patients and methods

This is a retrospective observational study involving two cohort groups of patients. Regarding the first cohort, of 101 patients admitted for COVID-19 infection from February 27 to April 28, 2020, at the Emergency Medical Ward of “Guglielmo da Saliceto” Hospital in Piacenza, Emilia-Romagna, Italy, 78 (77.2%) had been excluded due to the absence of ferritin evaluation, and 6 for negative SARS-CoV-2 swab. The remaining 17 patients diagnosed with COVID-19 pneumonia made up group A (11 males − 64%; 6 females, mean age 68.8). Regarding the second cohort, 30 patients (17 males − 56%; 13 females, mean age 66.2) with double negative nasopharyngeal swab for SARS-CoV-2 admitted from August 25 to September 15, 2020, to the Emergency Surgical Ward of “Guglielmo da Saliceto” for acute surgical disease composed group B. There were no comorbidity differences between the two groups (A = 2; B = 2.7) in terms of age over 65, diabetes, cardiovascular events, hypertension, obesity, kidney disease, and previous cancer history.

In group A, all patients were positive for 2019-nCoV by real-time polymerase chain reaction from the nasopharyngeal swab [5] and had been investigated upon admission with bedside lung ultrasound and chest CT scan, which demonstrated interstitial pneumonia at different stages. White blood count and serum values of C-reactive protein (CRP) (nv 0–0.5ma/dL) and ferritin (nv 12–300 ng/mL) were quantified in all patients enrolled in the study. Data collected were analyzed to check differences in the two groups—COVID-19 versus non-COVID-19, particularly to understand the relationship between the inflammatory processes and serum ferritin levels in COVID-19 patients.

In our study, we ascertained the hypothesis of serum ferritin as a marker of SARS-CoV2 infection, which could be a simple and useful laboratory test to identify and monitor the inflammatory process in COVID-19 patients.

## Results

In group A, a total of 101 patients were hospitalized in the Emergency Ward. 17 (16,8%) of them died during hospitalization, 21 (20,7%) were transferred to other hospitals due to either the worsening of the respiratory failure or the lack of beds in our Intensive Care Unit, while 63 (62%) were discharged home. Only 23 (22,7%) of them had their serum ferritin measured during hospitalization, 6 of which were excluded due to the absence of positive SARS-CoV-2 swab.

The median (interquartile range) serum ferritin value was 674 (1284) ng/mL, and it was double the cutoff (300 ng/mL) in 9 of 17 patients (52%). Levels in deceased patients were 1981, 1563, 239, 7438, and 344 with a median (IQR) value of 1563 (1637); in patients transferred to continue treatment in other hospitals: 110, 219, 1903, and 3194, with a median value of 355 (1032); and in patients discharged at home: 279, 674, 790, 906, 726, 462, 2181, and 72, with a median value of 700 (761).

In group B, a total of 30 patients admitted for an acute surgical problem were examined. Patients characteristics were as follows: six patients had acute appendicitis, one perforated peptic ulcer, three colonic perforations, two neoplastic intestinal obstructions, eight intestinal obstructions that did not require surgery, three acute cholecystitis, three acute diverticulitis Hinchey 1, two obstructed hernia, one perianal abscess, and one traumatic splenectomy. Iron status was evaluated once in 20 patients, twice in 5 patients, three times in 2 patients, and four times in 3 patients, with a total of 48 blood samples. In this group, ferritin levels were increased in 13 out of 48 blood samples (27%), with a top value of 804. High white blood cell count and CRP (cutoff > 5 mg/dL) were present in 20 and 24 samples respectively. Leucocytosis was present in 16 patients and, in this subgroup, simultaneous augmented ferritin was present in 3 out of the 16 cases (18%), while CRP was over the cutoff in 10 out of 16 (62%). The median (IQR) value of ferritin level in total blood samples of group B was 231 and, in the subgroup with leucocytosis, 149 (145).

**Fig. 1:**
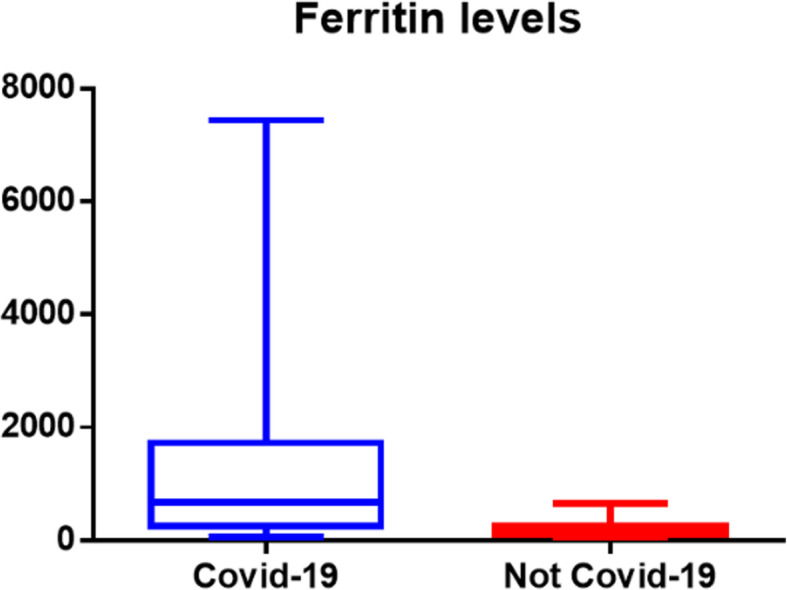
Median interquartile levels of COVID versus not-COVID patients

**Fig. 2:**
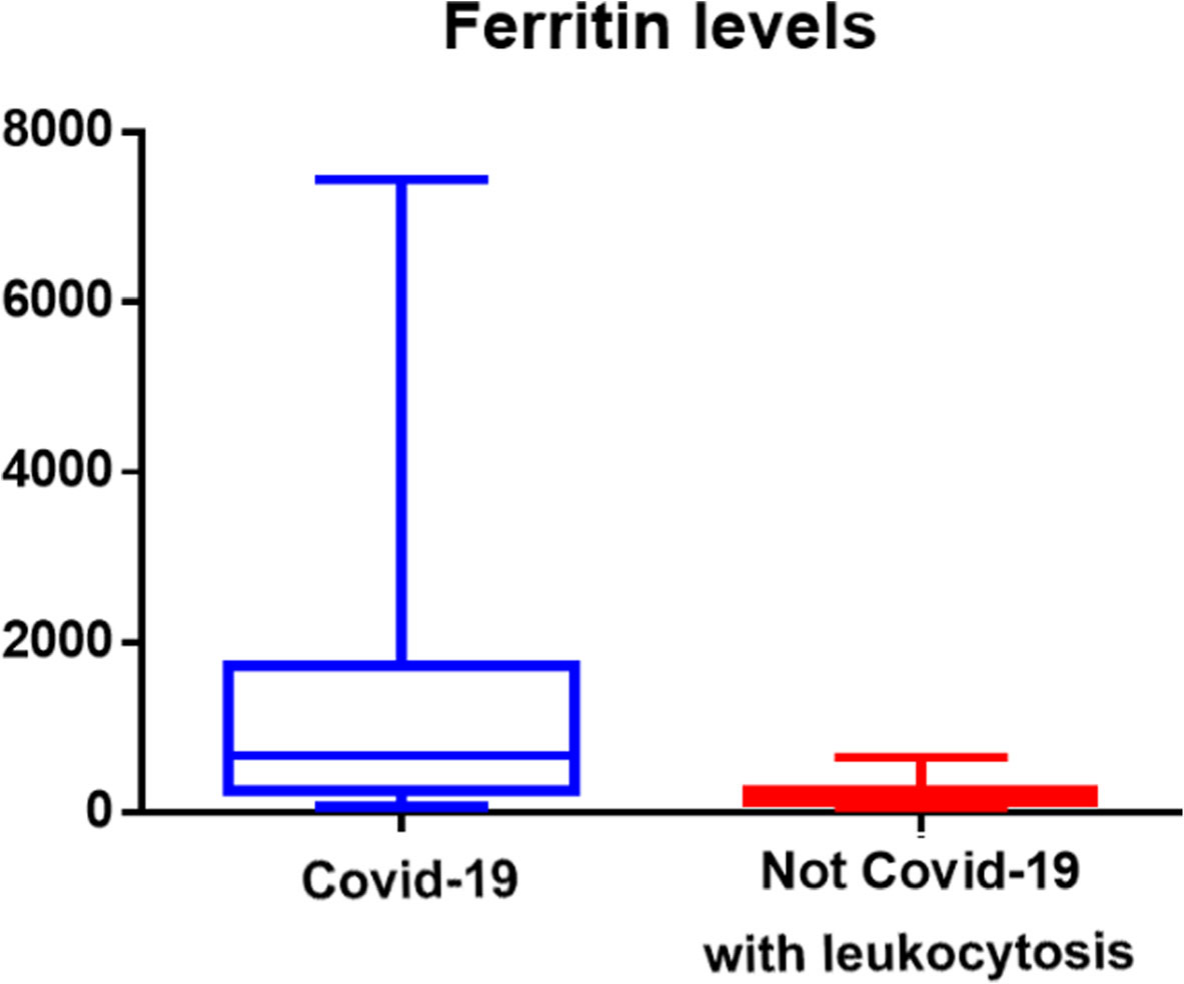
Median interquartile levels of COVID versus not-COVID with leucocytosis

**Fig. 3:**
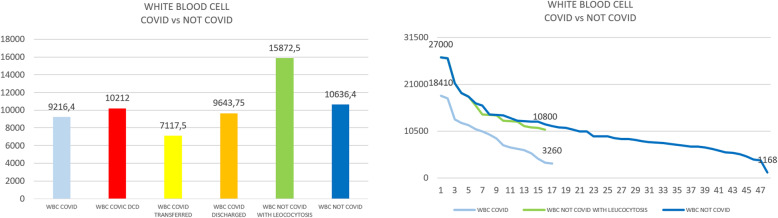
**a** Arithmetic mean of white blood cells in COVID versus not-COVID. **b** Scaling distribution of white blood cell in COVID versus not-COVID

**Fig. 4:**
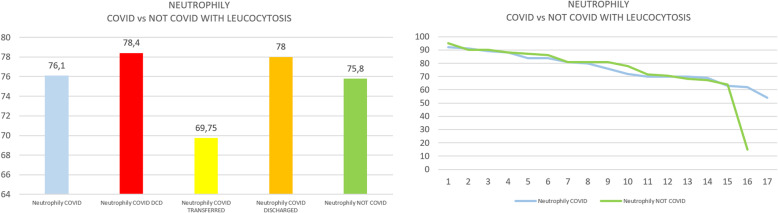
**a** Arithmetic mean of neutrophily in COVID versus not-COVID. **b** Scaling distribution of neutrophily in COVID versus not-COVID

**Fig. 5:**
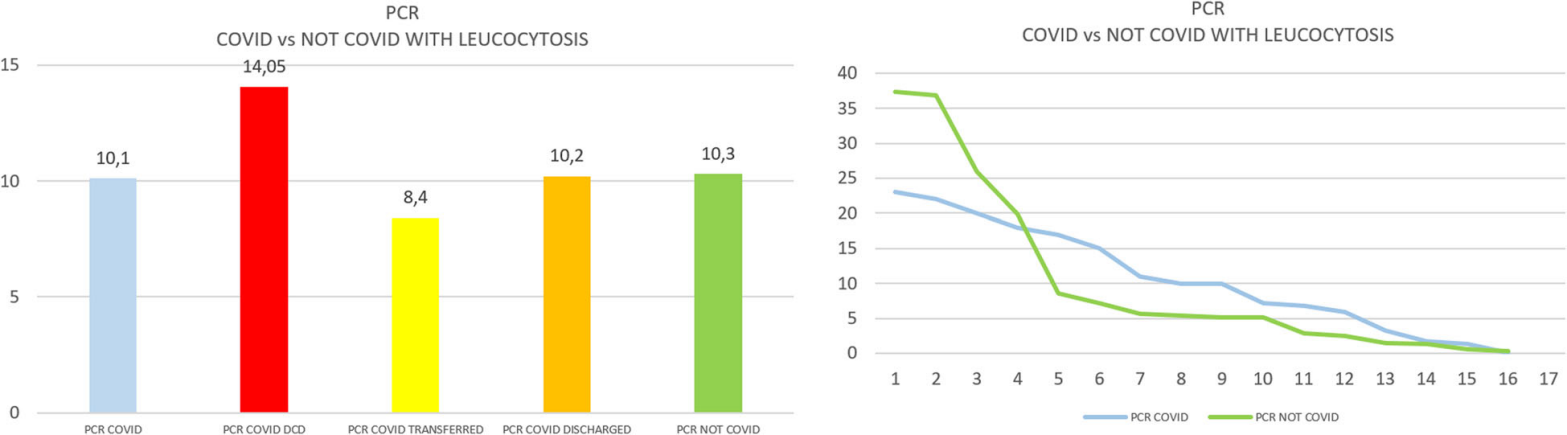
**a** Arithmetic mean of C-reactive protein in COVID versus not-COVID. **b** Scaling distribution of C-reactive protein in COVID versus not-COVID

**Fig. 6:**
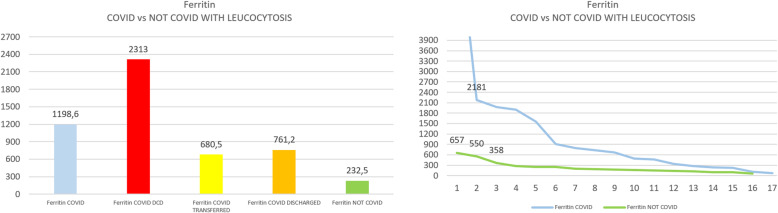
**a** Arithmetic mean of Ferritin in COVID versus not-COVID with leucocytosis. **b** Scaling distribution of Ferritin in COVID versus not-COVID with leucocytosis

Group A showed a significantly higher median level of ferritin in comparison with the entire group B (two-tailed Mann-Whitney test, *p* < 0.0001), even when also compared to the subgroup with leucocytosis (p< 0.0014) (see Figs. 1 and 2). Further analysis of group B was done in patients with leucocytosis, using only the corresponding maximal values of leucocytes registered for each patient compared to group A (see Figs. 3, 4, 5, and 6)

In group A, ferritin levels were over the cutoff value in 12 out of 17 (70.5%), whereas in group B, it occurred only in 27% of cases and in 18% of cases when leucocytosis was also present. Moreover, an extreme discrepancy in ferritin values was present between the two groups. In group B, when ferritin was increased, it exceeded two times the cutoff only in 3 out of 48 samples (6%), compared to 9 out of 17 patients (56%) in group A.

Finally, incredibly high ferritin levels were present in group A, with up to 24 times (7438) or 7 times (2181) the cutoff.

## Discussion

When an infection occurs, the inflammation process goes through SIRS (systemic inflammatory response syndrome), then SEPSIS, and subsequently SEVERE SEPSIS. The International Sepsis Definitions Conference [1] defined the criteria for SIRS as the presence of two or more of the following parameters: body temperature > 38 °C or < 36 °C, heart rate > 90 beats/min, respiratory rate > 20 breaths/min (or arterial pCO2 < 32 mmHg, indicating hyperventilation), white blood cell count > 12.0 × 109/L or < 4.0 × 109/L (or > 10% immature forms), sepsis = infection + SIRS, and severe sepsis = sepsis + evidence of organ dysfunction.

The parameters commonly used to monitor inflammation process and sepsis evolution are lactate, C-reactive protein, procalcitonin, and, more recently, TNF, IL-1beta, and IL-6. The mortality rate remains high (30%), revealing that our knowledge of sepsis is not well understood, and there is no specific treatment yet [6].

Although ferritin is reported as an acute-phase protein, there is literature lacking in reporting the particular modified levels, leading to a misunderstanding regarding its interpretation [7]. Recently, the role of IL-6 has become a key marker in COVID patients, and the classical parameters used for sepsis score do not clarify or answer some of the questions posed by the cytokine storm. The link between IL-6 and iron metabolism is well known [8], but iron parameters are not yet considered a standard biomarker to monitor septic evolution. Even in most recent guidelines for COVID (COVID-19) [2] or in reports about sepsis [9], there is no mention of ferritin, transferrin, or other iron parameters. However, in a recent prospective analysis presented by Brandtner [10], iron parameters such as ferritin and transferrin saturation levels both correlate with SOFA score (Sequential Organ Failure Assessment) with a significative value: *p* = 0.043 and *p* = 0.034, respectively.

As mentioned in our study, the inflammatory process we are looking for seems different in the usual acute inflammation like emergency surgery than it appears in COVID patients. In patients admitted for acute abdomen or other surgical pathology, the acute inflammatory process correlates with the standard parameters but not with ferritin modification. On the contrary, in COVID patients, iron modification seems to occur immediately. In a recent meta-analysis published by Zeng [11], ferritin had been considered only in 4 of the 16 studies analyzed, but he underlined that ferritin levels could classify COVID patients’ severity. This reflects the fact that very few studies at the moment consider iron metabolism in COVID and non-COVID patients. To emphasize the opposite, we cite as an example one of the patients described in our study, who was admitted for acute peritonitis due to rectal perforation, with WBC of 27,000, neutrophil count 90%, CRP of 36, and with a ferritin level of 657, which was lower than the median value among discharged patient in COVID group.

It is necessary to dig deep into ferritin formation in order to gain more knowledge regarding the processes that are comprised, where IL-6 and other factors play a significant role. Ferritin production occurs when intracellular iron concentration augments, with iron being stored in the form of ferritin and subsequently expelled from the cell. Intracellular iron accumulation occurs in two main modalities: hyperexpression of Transferrin receptor 1 which internalizes transferrin, and hepcidin expression. Hepcidin inhibits iron ions expulsion blocking ferroportin, which is the only siderophore of the cells.

Interestingly, in a recent publication of Jiang [12] about patients admitted in ICU, high hepcidin levels were directly related to mortality with a superior predictive value as well as a high specificity compared to other inflammatory parameters. Furthermore, as Kell explained in 2014, ferritin is not only related to the inflammation process but could be a direct indicator of cellular damage, especially when its value is over 600 ng/mL [13], implying a direct relationship between organ damage and ferritin formation.

The hyperactivation of this process causes cell death by so-called ferroptosis. This latter becomes one of the mechanisms described in acute respiratory distress syndrome (ARDS), quite similar to COVID pneumonia.

Following these observations, we might suggest that the usual inflammatory process starts with the classical SIRS-SEPSIS and subsequently affects iron metabolism. In contrast, in COVID patients, iron modifications occur as a first step, followed by SIRS, and likely resulting in a sort of severe sepsis. Furthermore, ferritin could be evaluated in surgical patients with negative SARS-CoV-2 swab as an indirect marker, in order to verify if they have been affected by COVID disease during their hospitalization, and as an indicator of severe sepsis at the same time.

Considering the lack of data presented in our study in the Group of COVID patients, we cannot confirm with statistical significance a direct correlation between COVID severity and ferritin levels, even though we could suggest it as supported by most recent literature data. On the contrary, we may confirm the absence of hyperferritinemic syndrome at the beginning of the usual inflammatory process and assess which values could be related to this phlogistic reaction phase.

Recent literature advocates hyperferritinemic syndrome as one of the main modifications in COVID infection [14–19], suggesting evaluation of ferritin levels as a parameter of infection. Presently, excluding reviews and metanalysis, only ten papers have been published with the contemporary topic of “ferritin” and “COVID” [20–29]. Moreover, we have suggested the evaluation of ferritin in COVID infection in our previous reports [30, 31]. This lack of literature reflects the low usage of iron parameters in common practice as revealed from our analysis in group A, which occurred at the beginning of the pandemic spread in Italy. Also, as Bataille [24] presented on 22 patients, this parameter can become useful in asymptomatic patients, and be used as a possible discriminant between COVID and non-COVID pneumonia, as posted by Garanti [29].

This study has several limitations, in particular, the narrow number of patients enrolled mainly due to an unusual serum ferritin testing in the initial period as well as its retrospective nature. Therefore, a wider analysis would be necessary in order to prove our outcomes.

## Conclusions

Even if ferritin is considered an acute-phase protein, its role in monitoring inflammation is still not very clear and, for this reason, it is not used routinely. The role of iron metabolism in COVID infection is increasing even without an accurate interpretation, and our study seems to confirm this observation. In the acute inflammation of surgical patients admitted for a procedure, and probably in other classical inflammation processes, iron modifications appear to be self-limited. However, our finding suggests the use of ferritin values as a marker for COVID infection.

## Data Availability

Separate files with anonymous content data are uploaded with the manuscript submission.

## Abbreviations

CRP: C-reactive protein
nv: Normal value
IQR: Interquartile range
SIRS: Systemic inflammatory response syndrome
SOFA: Sequential Organ Failure Assessment
ICU: Intensive care unit
WBC: White blood cell
ARDS: Acute respiratory distress syndrome
